# Understanding household and food system determinants of chicken and egg consumption in India

**DOI:** 10.1007/s12571-023-01375-3

**Published:** 2023-07-17

**Authors:** Lavinia Scudiero, Mehroosh Tak, Pablo Alarcón, Bhavani Shankar

**Affiliations:** 1 Veterinary Epidemiology, Economics and Public Health Group, Department of Production and Population Health, Royal Veterinary College, North Mymms, Hawkshead Lane, Hatfield, Hertfordshire, AL9 7TA UK; 2 Institute for Sustainable Food, The University of Sheffield, Sheffield, UK

**Keywords:** Poultry, India, Chicken and eggs consumption, Animal sourced food, Consumption determinants, Food systems

## Abstract

Poultry is one of the fastest-growing agricultural sectors in India and its demand is said to be rising. There is a perception that higher incomes, growing population, urbanisation, and increased productivity in the industry have influenced Indian poultry consumption. However, consumer surveys have shown that the average poultry consumption in India has remained low. With this in mind, the paper analysed household determinants of chicken and egg consumption within the Indian population, using two rounds of National Sample Survey data (1993–1994 and 2011–2012). By conducting a spatiotemporal analysis of household consumption and expenditure survey and by using truncated Double Hurdle and Unconditional Quantile regressions (UQR) models, this study explored socio-economic and food system determinants of chicken and egg consumption in India. Key results highlight that while consumption has increased marginally over twenty years, supply-side determinants, such as price and poultry production concentration, influenced heterogenous consumption patterns in India. We also find evidence that historically marginalised groups consumed more chicken and eggs in comparison to non-marginalised groups and preliminary evidence suggests how household gender dynamics influence different consumption patterns. Adequate consumption of poultry is important to improve nutrient-deficient diets of vulnerable groups in India. Our findings on demand side determinants of poultry products are crucial to support consumer tailored actions to improve nutritional outcomes along with the Indian poultry sector policy planning.

## Introduction

India's economic growth and social improvements in recent decades have co-existed with the persistent double burden of malnutrition. Nutrient deficiencies and undernutrition have been among the top risk factors for stunting, death, and anaemia in the country (Das, 2016; Maitra et al., 2013). The inclusion of appropriate levels of animal source foods (ASFs) in diets to improve dietary quality and nutritional outcomes of the population, especially those of children in low resources countries, is highlighted in several studies (Adesogan et al., 2020; Iannotti et al., 2014; Zaharia et al., 2021; Zhang et al., 2016). Among ASFs, eggs and chicken meat are particularly relevant due to their nutrient-rich characteristics and relative affordability compared to other animal-origin nutrient-dense foods (Iannotti et al., 2014; MAFW, 2017).

While there is a perception that consumer demand for poultry products in India is rising due to changes in the relative price of poultry, higher incomes, and the vertical integration of the poultry industry, the average per-capita consumption for chicken and eggs has remained low (Bruckert, 2021; Devi et al., 2014; MAFW, 2017). The inclusion of eggs and chicken meat in diets is recommended in the Indian dietary guidelines (National Institute of Nutrition, 2011). However, recent estimates from the Ministry of Agriculture and Farmers' Welfare (MAFW) show that the per-capita consumption in India is only 69 eggs and 3.35 kg of chicken per person per annum (MAFW, 2017). India also ranked as one of the countries with the lowest prevalence of egg consumption among children less than 24 months of age compared to other countries in South Asia. With data from 2004–2011, the prevalence of egg consumption among children in South Asia was more than 28%. While in Bangladesh in 2011 the prevalence was at 25%, in India only 5% of the children consumed eggs in 2007 (Iannotti et al., 2014). The per-capita consumption of egg and chicken is particularly low amongst rural Indians who consume half of what their urban counterparts do (MAFW, 2017). According to official estimates, the per-capita consumption over 30 days for chicken was 178 g in rural India and 239 g in urban India, and for eggs only 1.94 in rural India and 3.18 in urban India (GOI, 2014). Possible causes for inadequate consumption include inequalities in regional prices and supply, low income, geographical inequities, and social differences (Landes et al., 2004; Pica-Ciamarra & Otte, 2010; Sharma et al., 2020). However, the evidence on the drivers of consumption is limited.

Rapid advancements and developments in the Indian poultry sector’s structure and operations have kept poultry products affordable and available in the southern states (ICFA, n.d.; Pica-Ciamarra & Otte, 2010). The private sector in Southern India has undergone expansion and the industrial chicken meat and eggs sector grew at an average annual growth rate of 9% and 6% from 2000–2001 to 2018–2019 (DAHD, 2020). On the demand side, Landes et al. (2004) found that, in southern India, per-capita poultry consumption has been higher in urban settings because higher-income consumers live in cities and because retail poultry prices offered by large-scale poultry production are significantly lower in the region’s urban areas (Landes et al., 2004). This implies that income and household characteristics might be important factors for consumption.

However, production facilities also impact demand. Indian consumers prefer to buy from live bird markets due to the perception that fresh poultry is of better quality. This perception is attributable to the short life of the poultry meat and the sector’s underdeveloped cold chains, refrigerators, and specialised equipment for the transport and conservation of live animals and poultry products (Devi et al., 2014; Pica-Ciamarra & Otte, 2010). Additionally, the high costs of transportation, shrinkage, and high rates of mortality pose difficulties in moving live birds over long distances from farms to rural areas. (Bruckert, 2015; N. Kumar & Kapoor, 2014; Lagos & Intodia, 2015; Pica-Ciamarra & Otte, 2010). This has led to a concentration of production and consumption processes in urban areas, neglecting rural ones (Nanda Kumar et al., 2022). Thus, research has suggested that supply-side factors are stronger determinants in the consumption of poultry products than demand-side factors (Pica-Ciamarra & Otte, 2010).

Other studies have highlighted the role of changing lifestyles and heterogeneous food habits and consumption patterns for meat and eggs in India (Filippini & Srinivasan, 2019; Kumar et al., 2016; Saha et al., 2013). In recent times, there has been a shift from strictly plant-based diets toward diets containing greater amounts of animal protein, especially poultry (Filippini & Srinivasan, 2019). This is due to an increasing middle class that has more purchasing power and is gradually shifting away from symbolic reasons and ceremonial patterns of eating non-vegetarian foods (Bruckert, 2021). The availability, accessibility, palatability and apparent innocuousness of chicken, make it a more accepted meat than beef or pork in India. Its production, acquisition, and consumption are no longer ritualised activities (Bruckert, 2015; Devi et al., 2014). However, food intake and dietary diversity can differ among social classes and across Indian regions (Choudhury et al., 2020; Sharma et al., 2020; Tak et al., 2019). Simoons (1994) noted that cultural preferences and values like religion and vegetarianism are important factors that limit the demand for poultry in the country (Simoons, 1994). The caste system influences local food eating and dictates acceptable and unacceptable cultural practices in Indian society (Dolphijn, 2006; Sathyamala, 2019). Thus, meat consumption, which is considered an “impure” practice by Hindus, is generally higher among Muslims, Christians, and tribal caste groups (Pingali & Khwaja, 2004). Whereas upper castes Hindus and political groups representing their values have been found to constrain meat and poultry products consumption in West and North India (Ahmad, 2018; Morris et al., 2018).

While chicken meat is generally not heavily associated with religious practices such as beef and pork consumption, its demand can fluctuate due to religious and cultural obligations practiced during certain days and weeks of the year (Devi et al., 2014; Lagos & Intodia, 2015). For example, during the Hindu festival of Navaratri (September—October for nine days), auspicious months, and mourning periods, chicken meat is avoided. Consumption is interdicted also when women are menstruating. Instead, during weekends, festivals and celebrations such as Christmas and New Year’s Eve, demand can be higher (Bruckert, 2021; Lagos & Intodia, 2015). Egg consumption decreases in summer due to a belief that it produces more body heat (heating foods are considered impure foods by Upper caste Hindu groups) and its perceived increased risk of poultry diseases during the season of hot temperatures (Khan & Ravichandran, 2015; Lagos & Intodia, 2015).

This work investigates the consumption of chicken and eggs and how it remains regulated by socio-economic aspects, despite processes of intensification of production where poultry products have become more available and affordable. Existing studies primarily include poultry as one of many food categories in broader food insecurity and poverty analysis discussions. Only a small body of literature explores determinants for poultry demand in Low-and Middle-Income Countries such as India, where production practices are intensifying yet malnutrition is persistent. Combining a focus on poultry production factors and specific household characteristics, the paper analyses economic, socio-demographic, cultural, and food system factors contributing to chicken and egg consumption in India. The study addresses this aspect by conducting a spatiotemporal analysis of household consumption and expenditure survey (HCES) data and by using truncated Double Hurdle and Unconditional Quantile Regressions (UQR) models.

Through this analysis, the paper contributes to existing literature on ASF consumption in two ways. First, using large scale nationally representative household consumption data, the paper provides an in-depth socio-demographic analysis of poultry consumption in India, highlighting that income, culture, household location, and intrahousehold dynamics may be important barriers to consumption. Second, the paper provides empirical evidence on heterogenous and unequal protein intake through chicken and eggs. The findings have policy implications for malnutrition, especially in the context of a high growth poultry industry as this sectoral growth may not directly translate to improved nutrition for all.

## Materials and methods

### Data

The paper analyses HCES data from the 50^th^ and 68^th^ rounds of the nationally representative cross-sectional National Sample Survey (NSS) conducted in 1993–1994 and 2011–2012. Although the latest available data is not recent, the NSS is considered the most reliable consumption level dataset in India (Ahmad, 2018). It is the largest available source of nationally representative data and is often used to analyse consumption trends in India (Aleksandrowicz et al., 2017). The two NSS datasets allow exploration of spatiotemporal heterogeneity in the consumption of chicken and egg across urban and rural areas, class and social groups. Data on the purchase and consumption quantities of different food items over a recall period of the last 30 days is used for this analysis. The original sample size consisted of 115,360 observations for the 50^th^ round and 101,662 observations for the 60^th^. The paper discarded outliers in the data in the form of households with extreme values of poultry consumption (eggs and chicken) per-capita per month (PCPM). Based on PCPM intake, outliers were calculated corresponding to households’ PCPM intake with values beyond 6 and 20 kg for chicken and 80 number for eggs for both rounds. Respectively, only 6 and 4 observations were excluded. The final sample sizes consist of 115,354 and 101,658 households.

### Methods

The paper performs data visualisation in the form of summary statistics, and temporal and spatial analysis of chicken and egg consumption distribution at the country and state levels. In order to compare ASF intake, we estimate the monthly average quantity purchased of all ASF apart from poultry. We then show the consumption distribution using the full set of observations. Then, the distribution restricted to the population that consumes chicken and eggs is presented. With the full set of observations, we also perform local polynomial smoothers to assess non-linear bivariate relationships between PCPM chicken and egg consumption and monthly per-capita expenditure (MPCE), as a proxy for household income, and fractional polynomials to assess the association between PCPM consumption and unit value, subject to regional influences, as a proxy for chicken and egg price. Subsequently, we carry out regression analyses to estimate determinants for household chicken and egg consumption. We test whether a set of determinants affected participation and level of consumption decisions across two decades. We then assess how the same set of determinants of consumption varied across the chicken and eggs PCPM consumption distribution only with the latest available data as the main determinants of consumption did not change in the Double Hurdle model. Data analyses are undertaken using Stata v.14 (StataCorp, 2015).

#### Variables

As outcome variables, we create two variables to represent household PCPM consumption of chicken and household PCPM consumption of eggs (PCPM kg and number respectively). Following the methodology used in existing studies on household consumption and expenditure data, per-capita consumption is calculated by dividing the total consumption of the items by the household size (Choudhury et al., 2020; Minocha et al., 2018; Smith & Subandoro, 2007). The urbanisation process has influenced consumers to consume food away from home and out of consumption has been gradually increasing in India (Deloitte, 2018; Pingali & Khwaja, 2004; Tefft et al., 2017). We thus apply adjustments that account for (i) the number of meals consumed away from home on payment, (ii) the number of meals received for free in the workplace, or at school or as assistance (NGOs etc.), or from other households (iii) and the number of meals prepared at home but consumed by non-household members. The following formula from the Nutritional Intake in India report (NSSO, 2014), is employed:

$$\mathrm{PCPM}= \mathrm{C} \mathrm{x} \left[\left(\mathrm{Mh} +\mathrm{Mf}\right) / \left( \mathrm{Mh} + \mathrm{Mg}\right)\right]$$

where: C is the derived quantity intake of the household as per the food item recorded in the schedule; Mh is the number of meals consumed by the household members in the household or received through purchase or as assistance or payment (excluding meals received from other households); Mf is the number of meals consumed by non-members; Mg is the number of meals received free from other households by household members.

The independent variables comprise socio-demographic indicators at the household level and food system factors associated with food consumption in India and other South Asian countries (Choudhury et al., 2020; Mehraban & Ickowitz, 2021; Minocha et al., 2018). These include MPCE (income), chicken and egg unit value (price), household size, presence of boys below eighteen years old in the household, gender and education level of the household head, agricultural employment of the household, urban location of the household, location of the household in major poultry producer states, and household religion and caste group. MPCE is calculated by dividing eggs and chicken expenditure values by the household size. Following the methodology used in existing studies, we estimate the unit values for eggs and chicken as a proxy for market prices by dividing household expenditure on eggs and chicken by the quantity purchased (this recognises that unit values incorporate a quality choice dimension)(Choudhury et al., 2020; Deaton, 1988; Minocha et al., 2018). For goods with different varieties and levels of quality, such as eggs and chicken, unit values reflect household choices about dietary quality in regard to types of produce consumed and relative consumption of produce quality grades (Choudhury et al., 2020). The unit values are subject to regional influences, we, therefore, take the average value at the state-region (1993–94) and district (2011–12) levels^1^ using weights. As poultry industrialisation has been concentrating in specific parts of the country, we create a variable that represents the major poultry producing states using available data from the Ministry of Agriculture for mid ‘90 s and 2012 (DAHD, 2012). Furthermore, as poultry backyard farming is part of subsistence farming, which accounts for 20% of the poultry market share in India (MAFW, 2017), we include a variable that represents whether the household’s primary employment is in agriculture.

Women have a determinant role in improving the diets of their families and research has shown that females allocate resources differently in the household compared to males (Amugsi et al., 2016; Hoddinott & Haddad, 1995; Kennedy & Peters, 1992). We thus include a variable that accounts for the female as head of the household. In addition, consumption might vary between children and adults thus, households with children below five years variable is also included. Evidence suggests that women and female children consume poorer diets in comparison to men and boys as reflected in a greater prevalence of undernourishment of various degrees and the lower growth dynamics of girls (Aurino, 2017; Lancaster et al., 2006; Raskind et al., 2018). To assess this, we include a variable representing households with boys below eighteen years. Education might play an important role in the demand for food products (Malhotra, 2013; Rautela et al., 2020). Therefore, we include a set of dummy variables to indicate the level of education for household heads as a proxy for knowledge.

We also include a set of dummy variables that represent the social and religious groups of the households as research suggested that social strata and religion are important determinants of food demand in India (Simoons, 1994). We use Schedule Tribes, Scheduled Castes, Other Backward Classes, and Other/Upper Castes as a classification for social groups,^2^ and Hindu, Muslim, Christian, Sikh, Buddhist and “other religions” for the religious group. Evidence suggests heterogeneity across states (Sharma et al., 2020; Tak et al., 2019). Therefore, state-level dummies are included. In addition, due to substantial evidence that in urban areas consumption is higher than in rural ones, we perform our analysis at the country level differentiating between rural and urban^3^ India (GOI, 2014; MAFW, 2017).

#### Empirical strategy

The study analyses determinants for household chicken and egg consumption measuring the association between PCPM consumption of chicken and PCPM consumption of eggs and the full set of independent variables. A high number of zero food expenditures on chicken and eggs in our dataset suggests that a censored regression that deals with zero consumption and missing observations model is suitable. Several models have been used to estimate this type of censored data, such as the Censored Tobit model, Heckman’s Selection model, and Cragg’s Double Hurdle (Balli et al., 2017; Burton et al., 1994; García & Labeaga, 1996; LUNG et al., 2020). Garcia and Labeaga (1996) describe three reasons for observing zeros in the dataset: 1) mismatch between purchase frequency and timing of the survey, 2) conscious consumption abstention, and 3) low purchasing power to consumption at the time of the survey. While NSSO data are collected across seasons and reason 1) might not apply to our case, we argue that zero consumption of eggs and chicken might occur due to reasons such as infrequent purchases because of income or non-participation because of important socio-cultural differences in ASF consumption habits in India, we thus test Double Hurdle models. In contrast to Tobit, Double Hurdle models recognise that the outcomes are determined by selection and level of use decisions and allow estimating first- and second-stage equations with sets of explanatory variables. We test the truncated Double-Hurdle model, which accounts for two independent decision paths to be taken about consuming (participation and expenditure/consumption level) against the lognormal and Tobit models for robustness. The tests reveal that the truncated double-hurdle model is the best econometric specification for the study. This implies that the allocation of consumption of chicken and eggs follows two independent decision paths: whether to consume or not and the second and, contingent on the first one, if the decision is to consume then how much in terms of quantity.

The model may be specified as:

- (i) observed expenditure/consumption
  $$\mathrm{y }=\mathrm{ d}.\mathrm{y}**$$
  where d = 1, if there is participation (w > O), = 0 otherwise;
- (ii) participation
  $$\mathrm{w }=\mathrm{ \alpha^\prime z }+\upmu$$
- (iii) expenditure/ level of consumption
  $$\begin{array}{c}\mathrm{y }**=\mathrm{max }\, (0,\mathrm{ y}*)\\ y* = \beta^\prime x + v\end{array}$$
  where w is the latent participation variable (consumption), and z and x denote the sets of regressors that influence participation and the level of expenditure respectively. It is assumed that the error terms, u and v, are randomly and independently distributed with a bivariate normal distribution.

Next, as the summary statistic highlights important heterogeneity in consumption distribution, we assess inequality in consumption looking into how the influence of the identified covariates on consumption varies across the chicken and eggs PCPM consumption distribution. To assess the contribution of determinants to changes in consumption, UQR, which is a method for identifying the distributional effects on outcomes in terms of changes in observed characteristics, is appropriate for the analysis. Specifically, we use Recentred Influence Functions (RIF) (Firpo et al., 2009; Rios-Avila, 2020). Unlike conditional quantile models, where only the conditional quantile effects of changes in explanatory variables are estimated, RIF allows estimating the unconditional quantile effects of the covariates on consumption at any quantile of the distribution providing unconditional estimates. These estimates effectively characterise the effect of a change in an explanatory variable in a population with different characteristics. The method has been applied in the analysis of food and nutrition outcomes by other researchers (Choudhury et al., 2020; Mishra et al., 2015; Zanello et al., 2016). We estimate UQR results for the 10^th^, 25^th^, 50^th^, 75^th^ and 90^th^ percentiles. Given that more than half of the observations report zero food expenditures for chicken and eggs, for our UQR analysis we exclude households that did not consume chicken and eggs in this analysis.

A RIF-regression is similar to a standard regression where the dependent variable, y, is replaced by the RIF of the quantile of interest.

These models take the form:

$$E\left[RIF\left(yi;q\tau | fy\right)Xi \right]=Xi\beta \mathrm{\gamma \tau } + \mathrm{ui}$$

where: y_i_ is the dependent (observed) variable, *q*_τ_ is the value of the outcome variable at the quantile_τ_, fy is the (unconditional) cumulative distribution function of Y_i.,_ X_i_ is a vector of independent variables, β is a vector of parameters to be estimated, γ represents the marginal impact of changes in the distribution of the explanatory variables on the quantile τ, and u_i_ is a vector of error terms.

## Results

### Descriptive results and nonparametric relationships

The descriptive data presented in Tables 1 and 2 confirm the low average consumption of various ASFs in India. Apart from an increase in the consumption of milk and eggs, there was a marginal increase in the consumption of all other ASFs during the two-decade period. The exception to this was chicken consumption which increased approximately 6 folds from 0.02 to 0.12 kg per month for rural areas and 0.03 to 0.17 kg per month for urban areas. Nevertheless, this increase was marginal overall. This is in line with recent literature analysing various food consumption in India (Sharma et al., 2020; Tak et al., 2019). Additionally, rural consumption of eggs rose from 0.71 to 1.33 eggs per month, while urban consumption increased from 1.69 to 2.2 eggs PCPM. Table 2 shows that the share of households consuming chicken and eggs increased by 29% and 14% across decades. However, even in 2011–12 56% of Indian households did not consume eggs, 60% did not consume chicken, and 44% did not consume both.

**Table 1:** Summary Statistics

	**1993–1994**		**2011–2012**	
	**Rural**	**Urban**	**Rural**	**Urban**
**Variable**	Mean	Mean	Mean	Mean
**Animal sourced food intake**				
Chicken (kg PCPM)	0.02	0.03	0.12	0.17
Egg (n PCPM)	0.71	1.69	1.30	2.20
Fish (kg PCPM)	0.20	0.21	0.22	0.21
Goat (kg PCPM)	0.05	0.09	0.03	0.05
Beef (kg PCPM)	0.02	0.02	0.03	0.03
Pork (kg PCPM)	0.008	0.004	0.006	0.004
Bird (1993–94:n; 2011–12: kg PCPM)	0.01	0.02	0.003	0.001
Milk (L PCPM)	3.92	5.09	4.47	5.71
**Household (HH) level indicators**				
Total meat expenditure (Rs PCPM)	5.61	9.87	45.65	62.89
Monthly per-capita expenditure	405.72	766.14	1,414.13	2,912.68
Chicken price (per kg in Rs)	36.74	40.10	122.65	125.28
Egg price (per egg in Rs)	1.15	1.18	3.81	3.65
HH size	5.20	4.81	4.60	4.05
Female headed HH (%)	9.71	10.35	12.08	11.73
HH with child < 5 years (%)	46.43	36.51	33.24	26.86
HH with boys < 18 years (%)	21.24	17.24	75.05	64.86
HH head is illiterate (%)	53.94	22.80	39.08	15.35
HH head has primary education (%)	11.93	13.16	13.15	10.77
HH head has secondary education (%)	9.69	14.85	15.14	14.45
HH head has university degree (%)	01.91	13.97	02.86	14.10
HH location (%)	73.46	26.54	68.75	31.25
Agricultural HH (%)	67.09	-	34.41	-
HH living in poultry producer states (%)	55.55	61.13	53.91	61.75
Hindu HH (%)	85.64	80.49	84.42	80.42
Muslim HH (%)	9.16	13.15	11.03	13.55
Christian HH (%)	2.29	2.87	2.19	3.04
Sikh HH (%)	1.73	1.53	1.59	1.35
Buddhist HH (%)	0.66	0.88	0.48	0.86
Other religion HH (%)	0.52	0.11	0.29	0.80
Scheduled Tribes HH (%)	11.20	3.38	11.35	3.55
Scheduled Castes HH (%)	22.21	13.76	21.22	14.18
Other Castes HH (%)	66.52	80.81	-	-
Other Backward Classes HH (%)	-	-	44.19	40.62
Other/Upper Castes HH (%)	-	-	23.22	41.64
Observations	69,191	43,894	59,693	41,965

**Table 2:** Share of households consuming and not consuming chicken and eggs

	**1993–1994**		**2011–2012**	
**Consumption**	**YES**	**NO**	**YES**	**NO**
	**HH**		**HH**	
Chicken	11%	89%	40%	60%
Eggs	30%	70%	44%	56%
Chicken and eggs	7%	67%	28%	44%
Observations	115,354		101,658	

**Fig. 1:**
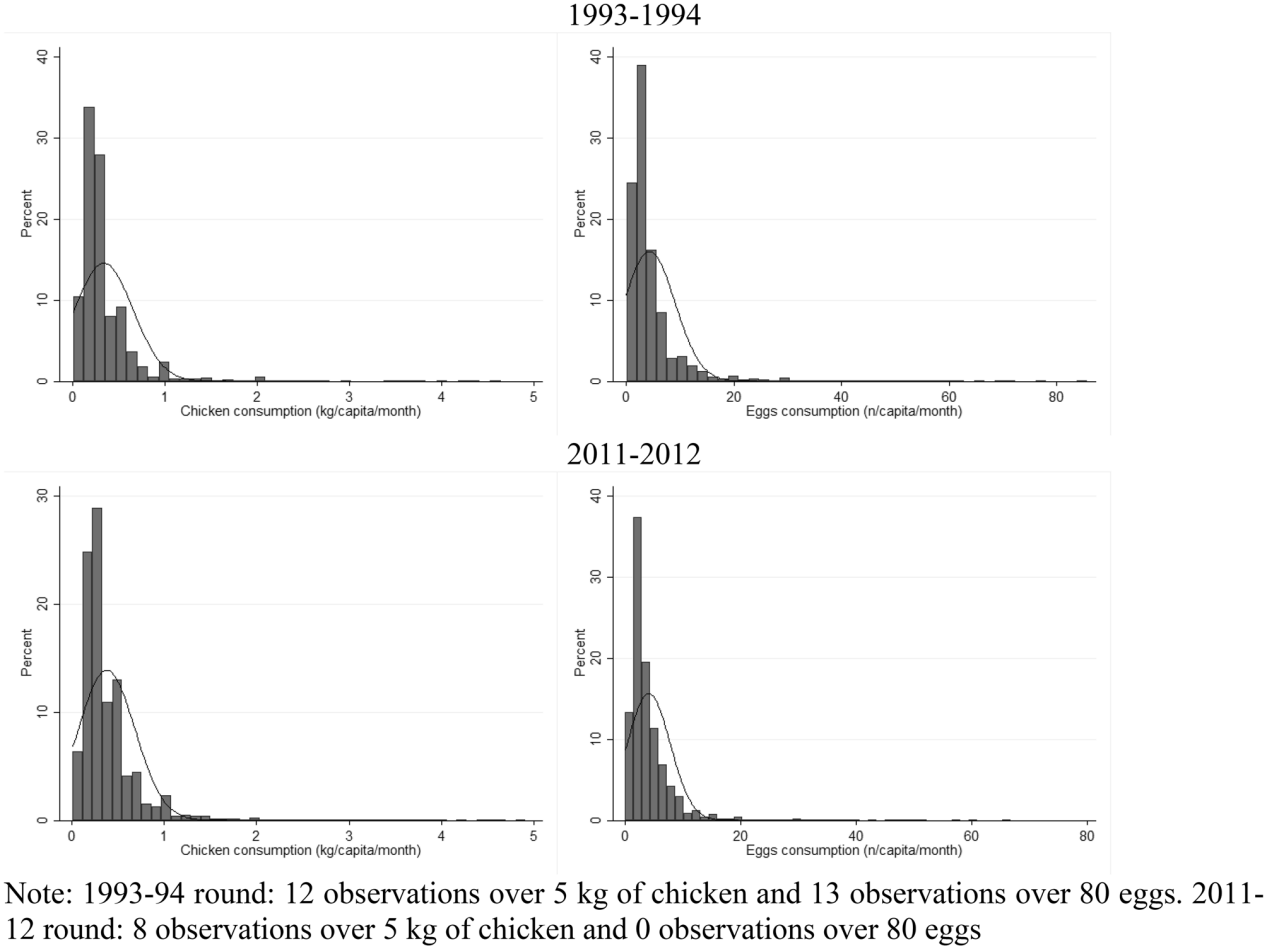
Distribution of consumption among households that consumed chicken and eggs**.** *Note: 1993–94 round: 12 observations over 5 kg of chicken and 13 observations over 80 eggs. 2011–12 round: 8 observations over 5 kg of chicken and 0 observations over 80 eggs*

Table 3 displays the quartiles of the distribution excluding the households that did not consume chicken and eggs for both periods. While we cannot appreciate an important consumption difference across twenty years, the tables show that PCPM intake differed across quartiles. Chicken meat consumption in 1993–94 ranged from 110 g PCPM in the first quantile to 280 g in the third, while egg consumption ranged from 1.23 to 3.38 PCPM in the third quantile. Similarly, in twenty years, chicken consumption increased from approximately 150 g of chicken and 1.5 eggs PCPM in the first quantile to almost 360 g of chicken and 4 eggs in the third quantile. In both periods, the average consumption doubled for both items from the third to the fourth quartile showing heterogeneity in consumption. Figure 1 further confirms that the PCPM chicken and egg consumption was highly unequal and heterogeneous, holding constant across decades. The distribution of chicken and egg consumption was concentrated at less than 0.5 kg chicken meat PCPM and less than 10 eggs PCPM. Table 4 incorporates a dietary quality perspective on chicken and egg consumption reflected in the unit value. It shows that in higher income quartiles, with higher prices, PCPM consumption was higher for chicken, while for eggs as PCPM consumption increased across quartiles prices were similar. However at higher price quartiles, for higher incomes there was lower PCPM consumption, particularly for eggs, potentially reflecting that a part of consumers with higher purchasing power consumed fewer products, but of better quality.

**Table 3:** Quantile distribution of households that consumed chicken and eggs

Chicken and egg consumption quartile distribution								
		**1993–1994**				**2011–2012**		
Quantile	(1)	(2)	(3)	(4)	(1)	(2)	(3)	(4)
**Chicken (kg PCPM)**								
Mean	0.11	0.18	0.28	0.61	0.14	0.23	0.36	0.70
HH (%)	20.06	23.02	27.97	28.94	22.38	23.42	27.18	27.03
Observations	2,438	2,797	3,399	3,517	9,174	9,601	11,143	11,083
**Eggs (n PCPM)**								
Mean	1 0.23	2.18	3.38	8.54	1 0.54	2.64	3.86	8.10
HH (%)	22.14	22.66	22.35	27.85	28.19	22.29	22.27	27.25
Observations	7,359	7,530	8,426	9,919	12,487	9,872	9,865	12,071

**Table 4:** Distribution of households that consumed chicken and eggs by monthly per-capita expenditure (income) and price quantiles

	1993–1994				2011–2012			
Quantile	(1)	(2)	(3)	(4)	(1)	(2)	(3)	(4)
**Consumption and price at monthly per-capita expenditure (income) quartiles**								
Monthly per-capita expenditure	156.54	236.47	333.26	714.61	739.28	1134.98	1673.85	3983.05
Chicken price (kg Rs)	43.55	43.94	45.03	47.91	117.35	120.73	121.43	125.79
Chicken consumption (kg PCPM)	0.21	0.26	0.31	0.47	0.24	0.33	0.41	0.55
Observations	3,042	3,040	3,040	3,029	10,251	10,250	10,251	10,250
Eggs price (n Rs)	1.18	1.21	1.23	1.27	3.71	3.58	3.50	3.59
Eggs consumption (n PCPM)	2.27	3.10	4.20	7.51	2.57	3.39	4.33	6.16
Observations	8,323	8,328	8,304	8,279	5773	6,972	7,551	8,380
**Consumption and monthly per-capita expenditure (income) at price quartiles**								
Chicken price (kg Rs)	32.72	41.54	48.87	62.66	91.45	116.55	124.76	156.07
Monthly per-capita expenditure	522.91	555.22	526.22	641.78	1769.35	1773.36	1875.98	2271.70
Chicken consumption (kg PCPM)	0.28	0.34	0.29	0.26	0.40	0.35	0.33	0.34
Observations	4,099	2,090	2,960	2,997	10,296	10,936	10,124	9,556
Eggs price (n Rs)	0.95	1.11	1.24	1.62	2.86	3.27	3.82	4.63
Monthly per-capita expenditure	424.94	423.47	458.37	492.60	1922.57	2014.53	1944.94	2004.39
Eggs consumption (n PCPM)	3.86	3.97	3.77	3.31	4.11	4.21	4.00	3.77
Observations	6,361	8,507	6,309	4,022	7,185	7,330	6,968	7,113

**Fig. 2:**
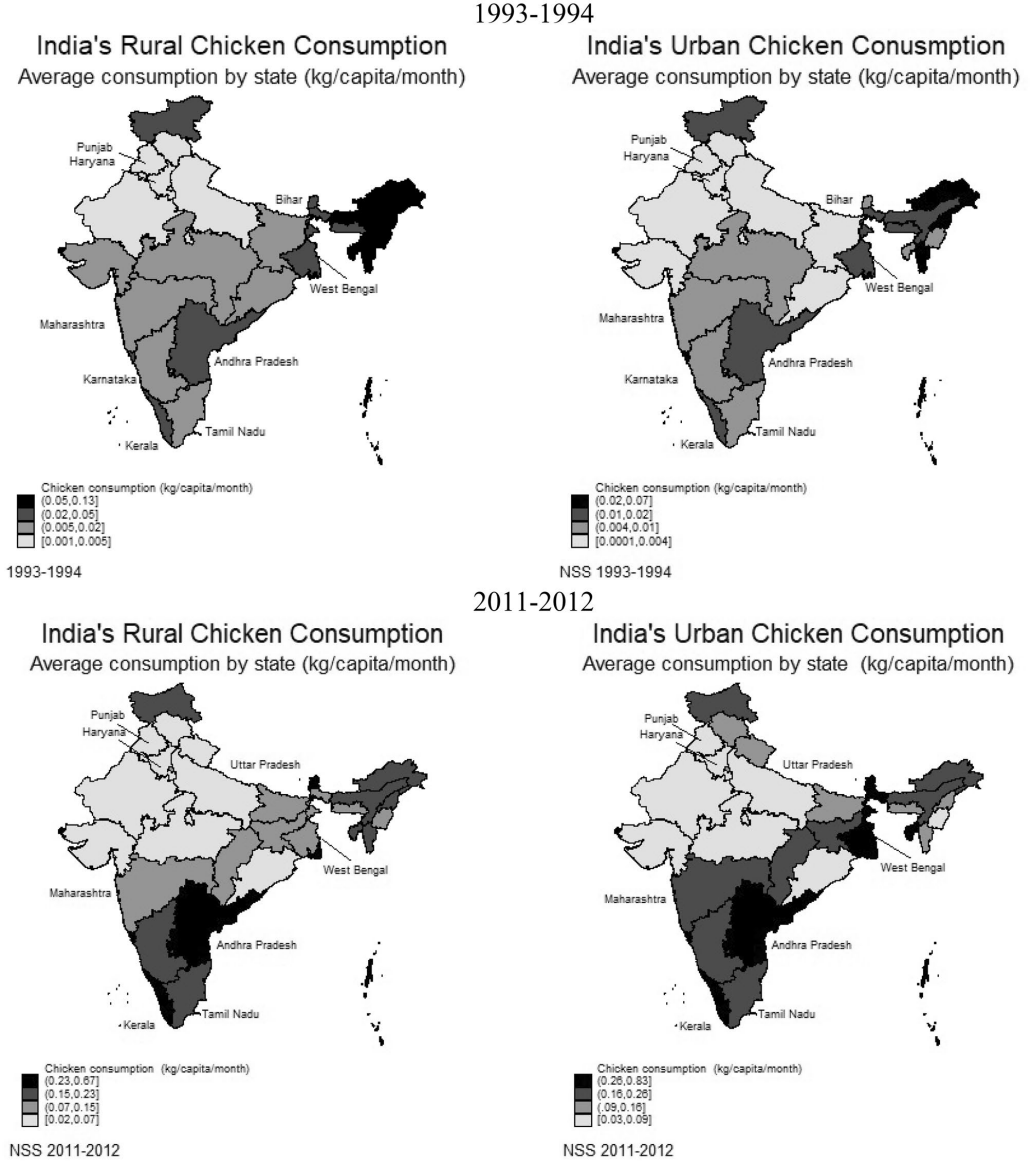

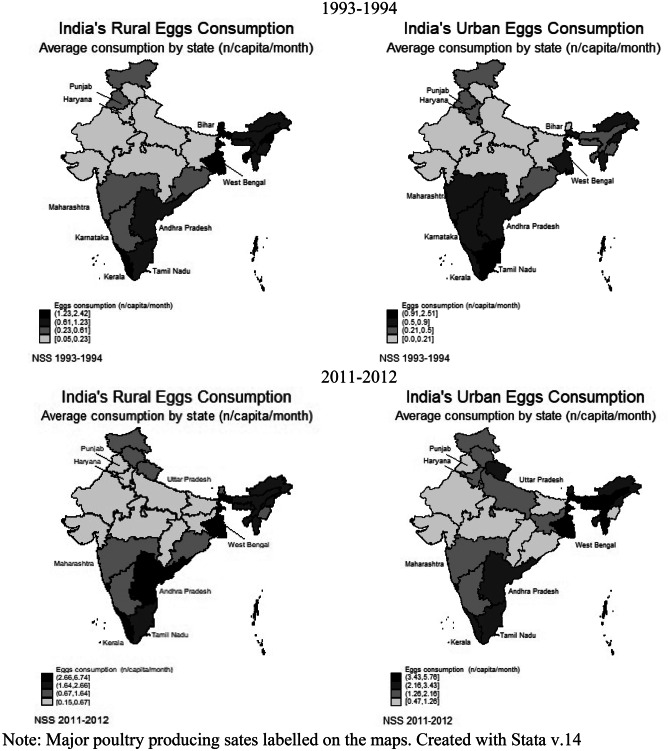
Rural and urban state-wise PCPM chicken (kg) and egg (n) consumption. *Note:* *Major poultry producing sates labelled on the maps. Created with Stata v.14*

Table 5 and Fig. 2 present spatial and temporal analysis of chicken and egg consumption across rural and urban areas. PCPM consumption was higher in the urban areas and southern and eastern states of India, whereas it was very low in North and West India. While in two decades there has been a marginal increase in consumption of eggs and chicken, in southern and eastern states PCPM consumption of both items has increased more substantially compared to the other zones. Figure 2 further shows that the consumption of chicken and eggs was higher amongst southern and eastern states. However, West Bengal also displayed high egg consumption that is not observed in Table 5, making it one of the major poultry consuming states, together with Tamil Nadu, Andhra Pradesh, and Kerala. This may be due to the concentration of production in southeast India that has made poultry products more accessible and cheaper in these regions (Landes et al., 2004; Pica-Ciamarra & Otte, 2010). Table 6 supports this. In twenty years, prices increased across India, but were lower in the south of India, particularly for chicken, compared to other regions. Furthermore, poultry producing states had prices slightly below the total price average at the national level.

**Table 5:** Average PCPM chicken (kg) and egg (n) consumption by region

	**1993–1994**				**2011–2012**			
	**Rural mean**		**Urban mean**		**Rural mean**		**Urban mean**	
**Region**	Chicken (kg)	Egg (n)	Chicken (kg)	Egg (n)	Chicken (kg)	Egg (n)	Chicken (kg)	Egg (n)
West	0.01	0.31	0.02	1.08	0.07	0.57	0.12	1.42
Observations	15,386	15,386	13,085	13,085	12,654	12,654	10,149	10,149
South	0.03	1.40	0.04	2.35	0.25	2.42	0.26	3.02
Observations	14,757	14,757	13,138	13,138	12,528	12,528	11,350	11,350
North	0.003	0.27	0.01	1.20	0.05	0.56	0.08	1.54
Observations	14,992	14,992	9,155	9,155	13,758	13,758	9,434	9,434
East	0.03	0.81	0.05	2.35	0.12	1.61	0.21	3.12
Observations	24,116	24,116	10,739	10,739	20,753	20,753	11,032	11,032

West: Rajasthan, Chhattisgarh, Madhya Pradesh, Gujarat, Daman and Diu, Dadra and Nagar Haveli, Maharashtra. South: Andhra Pradesh, Karnataka, Goa, Lakshadweep, Kerala, Tamil Nadu, Puducherry, Andaman and Nicobar. North: Jammu and Kashmir, Himachal Pradesh, Punjab, Chandigarh, Uttaranchal, Haryana, Delhi, Uttar Pradesh. East: Bihar, Sikkim, Arunachal Pradesh, Nagaland, Manipur, Mizoram, Tripura, Meghalaya, Assam, West Bengal, Jharkhand, Orissa.

**Table 6:** Average chicken (kg) and egg (n) price (Rs)

	**1993–1994**		**2011–2012**	
	**Chicken (Rs)**	**Egg (Rs)**	**Chicken (Rs)**	**Egg (Rs)**
**Variable**	**Mean**	**Mean**	**Mean**	**Mean**
India	37.64	1.16	123.47	3.76
**Region**				
West	36.44	1.30	126.41	3.89
South	40.43	1.01	117.83	3.21
North	43.84	1.32	129.35	4.05
East	30.57	1.01	120.81	3.91
Major poultry producing states	39.47	1.06	123.35	3.61
Non major poultry producing states	35.21	1.30	123.64	4.00

Table 7 shows that in both rounds, households that were Christian, Muslim, and Buddhist, on average, consumed more chicken and eggs PCPM than households that belonged to Hindu Sikh and other groups. Table 8 presents the PCPM consumption by social groups. Although consumption was very low across all groups, notably at the rural level, we observed that while with chicken there was consumption consistency across the groups, more disparities in egg consumption existed. Eggs PCPM consumption was lower among the marginalised castes and higher among Other/ Upper Castes for both rounds, especially at the urban level. Noteworthy, the chicken PCPM consumption of Other/ Upper Castes groups equalled or was slightly lower than the PCPM intake of the marginalised castes. Gains in poultry production and associated lower prices for middle-class consumers and higher vegetarianism trends among the upper castes might be the explanation for the similar chicken consumption patterns among strata groups.

**Table 7:** Average PCPM chicken (kg) and egg (n) consumption by religious groups

	**1993–1994**				**2011–2012**			
	**Rural mean**		**Urban mean**		**Rural mean**		**Urban mean**	
**Religion**	Chicken	Egg	Chicken	Egg	Chicken	Egg	Chicken	Egg
Hindu	0.01	0.63	0.03	1.59	0.12	1.18	0.16	2.08
Observations	56,021	56,021	35,362	35,362	45,603	45,603	31,458	31,458
Muslim	0.03	1.18	0.03	1.86	0.15	2.13	0.20	2.70
Observations	60,53	60,53	6,055	6,055	7,043	7,043	6,092	6,092
Christian	0.06	1.98	0.10	4.10	0.22	2.44	0.32	4.14
Observations	3,718	3,718	2,794	2,794	4,293	4,293	2,775	2,775
Sikh	0.01	0.51	0.04	1.75	0.04	0.40	0.10	1.16
Observations	1,643	1,643	934	934	1,314	1,314	702	702
Buddhist	0.01	0.79	0.01	1.35	0.16	1.39	0.19	2.17
Observations	658	658	326	326	737	737	357	357
Other religions	0.03	0.74	0.04	1.31	0.13	4.17	0.24	3.87
Observations	1,098	1,098	646	646	703	703	581	581

Other religions includes Other Religions, Jain and Zoroastrian.

**Table 8:** Average PCPM chicken (kg) and egg (n) consumption by castes

	**1993–1994**				**2011–2012**			
	**Rural mean**		**Urban mean**		**Rural mean**		**Urban mean**	
**Social group**	Chicken	Egg	Chicken	Egg	Chicken	Egg	Chicken	Egg
Scheduled Tribes	0.04	0.50	0.05	1.28	0.13	1.06	0.16	1.95
Observations	13,363	13,363	3,082	3,082	10,000	10,000	36,28	3,628
Scheduled Castes	0.01	0.57	0.02	1.36	0.11	1.32	0.18	2.13
Observations	13,001	13,001	5,285	5,285	10,194	10,194	5,502	5,502
Other castes	0,02	0.80	0.03	1.76				
Observations	45,778	45,778	37,724	37,724	-	-	-	-
Other Backward Classes	-	-	-		0.13	1.22	0.18	2.23
Observations	-	-	-		23,757	23,757	16,157	16,157
Other Upper Castes	-	-	-		0.12	1.55	0.15	2.22
Observations	-	-			15,733	15,733	1,6673	1,6673

**Fig. 3:**
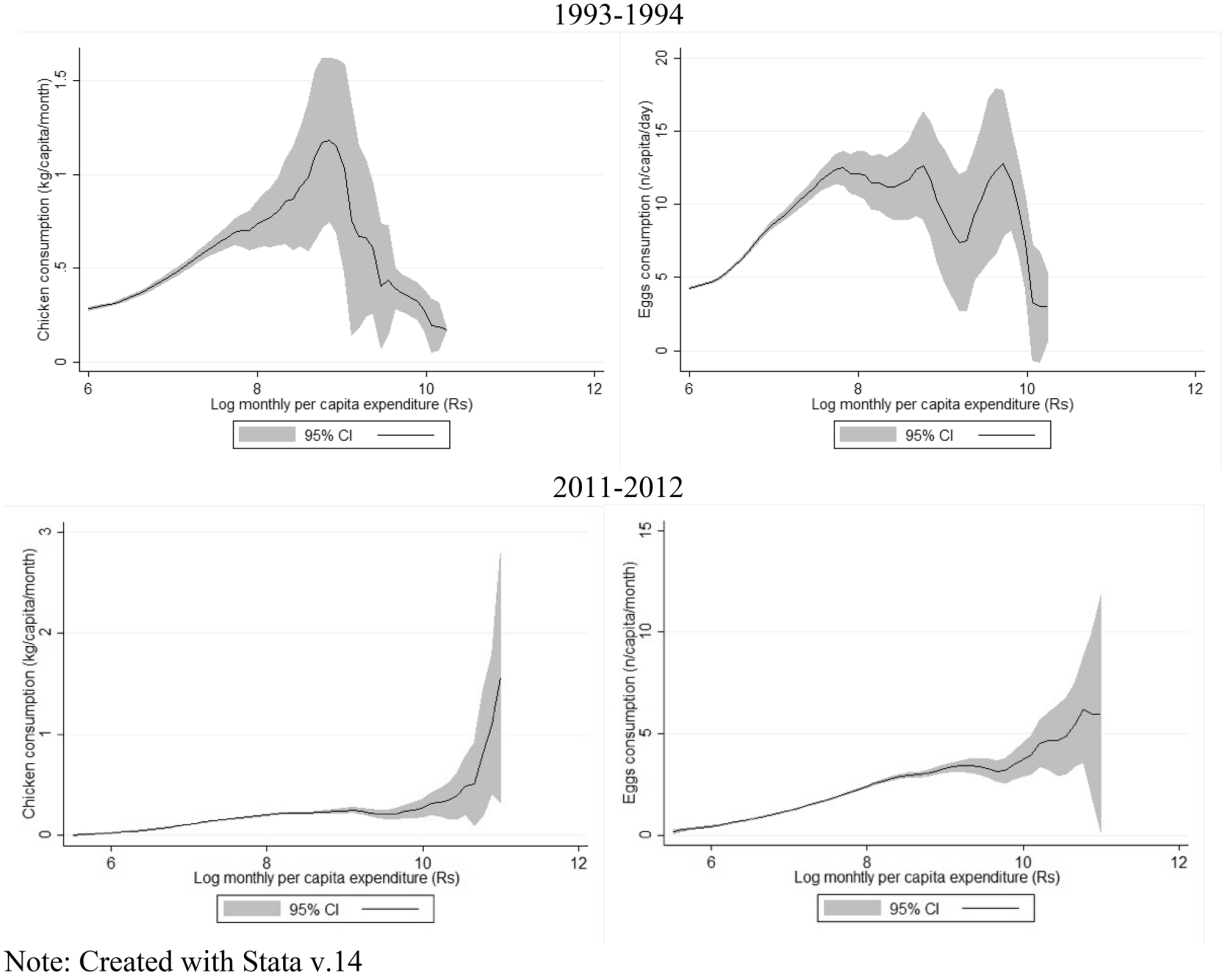
Nonparametric estimates of the relationship between chicken and egg consumption and monthly per-capita expenditure (Rs). *Note: Created with Stata v.14*

**Fig. 4:**
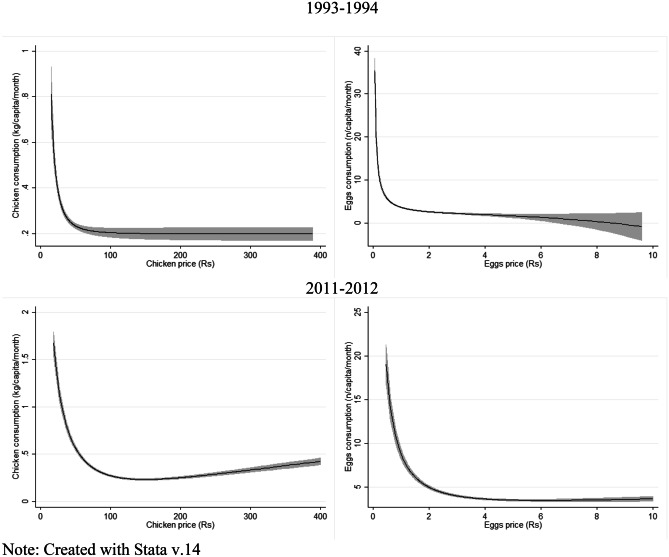
Nonparametric estimates of the relationship between chicken and egg consumption and price (Rs) per kg of chicken and number of eggs with region and district influences as fixed effects. *Note: Created with Stata v.14*

Figures 3 and 4 present the bivariate relationships between consumption and income and fractional polynomial associations between consumption and prices. In Fig. 3, in 1993–94, for the most part, a positive increase in consumption can be observed as the log of PCPM expenditure (income) increases, however, a drop in consumption is seen at higher incomes. In 2011–12 a constant positive increase in PCPM consumption for both chicken and eggs as income increases can be observed. Whereas Fig. 4, shows a downward trend in chicken and egg consumption as the prices of the items, controlled for regional influences, increased.

### Determinants for chicken and egg PCPM decision and intensity consumption

Next, Tables 9 and 10 present the results of our two-part model. It includes the full set of covariates (as described in Table 1). In column (1) the participation hurdle estimates the factors that influence the decision to consume, while in column (2) the intensity hurdle estimates the factors influencing the level (quantity) consumed.

**Table 9:** Truncated Double Hurdle Model Regression Estimates

**1993–1994**				
	**Column 1** **Consumption participation**		**Column 2** **PCPM consumption intensity** **Marginal effects**	
**Variables**	**Chicken (Kg)**	**Eggs (n)**	**Chicken (kg)**	**Eggs (n)**
Log monthly per-capita expenditure	0.56***	0.45***	0.41***	5.33***
	(0.01)	(0.01)	(0.01)	(0.06)
State-region price	-0.02***	-1.10***	-0.00***	-3.86*
	(0.00)	(0.02)	(0.00)	(0.16)
HH size	0.06***	0.05***	-0.05***	-0.33***
	(0.00)	(0.00)	(0.00)	(0.01)
HH head is female	-0.14***	-0.07***	0.07***	0.13
	(0.02)	(0.01)	(0.02)	(0.11)
HH has children below 5 years	0.01	0.05***	-0.06***	-0.22**
	(0.01)	(0.01)	(0.01)	(0.07)
HH has boys below 18 years	-0.01	-0.02**	0.03***	0.08
	(0.01)	(0.01)	(0.01)	(0.05)
HH head is illiterate	-0.12***	-0.17***	0.02	-0.40***
	(0.01)	(0.01)	(0.01)	(0.08)
HH head has primary education	-0.06***	0.04***	0.00	-0.12
	(0.02)	(0.01)	(0.01)	(0.09)
HH head has secondary education	-0.03	0.07***	-0.02	-0.22*
	(0.02)	(0.01)	(0.01)	(0.09)
HH head has university education	0.06**	-0.08***	0.03**	0.55***
	(0.02)	(0.02)	(0.01)	(0.12)
Urban HH	-0.13***	0.02	-0.10***	0.59***
	(0.01)	(0.01)	(0.01)	(0.08)
Agricultural HH	-0.00	-0.24***	0.02	-0.46***
	(0.01)	(0.10)	(0.01)	(0.09)
HH in poultry producing states	0.32***	0.29***	0.00	1.00***
	(0.01)	(0.01)	(0.06)	(0.07)
**Religion (Hindu as baseline)**				
Muslim HH	0.25***	0.49***	0.13**	2.01***
	(0.02)	(0.01)	(0.01)	(0.09)
Christian HH	0.25***	0.48***	0.00	1.23***
	(0.03)	(0.02)	(0.01)	(0.12)
Sikh HH	-0.24***	-0.42***	-0.07	-2.04***
	(0.04)	0.03	(0.04)	(0.28)
Buddhist HH	0.21***	0.18***	-0.02	0.99**
	(0.05)	(0.04)	(0.04)	(0.30)
Other religions HH	-0.40	-0.38***	0.15	0.46
	(0.03)	(0.03)	(0.03)	(0.36)
**Caste (Other Castes as baseline)**				
Scheduled Tribes HH	0.50***	-0.11***	0.17***	-0.21
	(0.02)	(0.02)	(0.20)	(0.11)
Scheduled Castes HH	0.02	0.12***	0.01	0.21
	(0.02)	(0.01)	(0.02)	(0.09)
State	YES	YES	YES	YES
Observations	115,354	115,354	115,354	115,354

Coefficients are shown, with standard errors in parentheses. *Significant at 5% level. **Significant at 1% level. ***Significant at 0.1% level. Other social groups included but not reported

**Table 10:** Truncated Double Hurdle model regression estimates

**2011-2012**				
	**Column 1** **Consumption participation**		**Column 2** **PCPM consumption intensity** **Marginal effects**	
**Variables**	**Chicken (kg)**	**Eggs (n)**	**Chicken (kg)**	**Eggs (n)**
Log monthly per-capita expenditure	0.37***	0.32***	0.30***	3.17***
	(0.01)	(0.01)	(0.00)	(0.04)
District price	-0.00***	-0.03**	-0.00***	-0.70***
	(0.00)	(0.01)	(0.00)	(0.08)
HH size	0.07***	0.04***	-0.02***	-0.28***
	(0.00)	(0.00)	(0.00)	(0.01)
HH head is female	-0.14***	-0.06***	-0.01*	0.20**
	(0.01)	(0.01)	(0.01)	(0.07)
HH has children below 5 years	-0.02**	0.02	-0.00	0.15**
	(0.01)	(0.01)	(0.00)	(0.05)
HH has boys below 18 years	0.21***	0.23***	-0.07***	-0.57***
	(0.01)	(0.01)	(0.01)	(0.06)
HH head is illiterate	0.06***	0.03**	0.03***	0.15**
	(0.01)	(0.01)	(0.01)	(0.06)
HH head has primary education	0.07***	0.07***	0.02**	0.03
	(0.01)	(0.01)	(0.01)	(0.07)
HH head has secondary education	0.06***	0.04***	0.00	-0.10
	(0.01)	(0.13)	(0.01)	(0.06)
HH head has university education	0.02	-0.14	-0.02**	-0.19**
	(0.02)	(0.02)	(0.01)	(0.08)
Urban HH	-0.04***	0.03**	-0.02***	0.20***
	(0.01)	(0.01)	(0.00)	(0.05)
Agricultural HH	-0.08***	-0.10***	-0.00	-0.22***
	(0.01)	(0.10)	(0.01)	(0.06)
HH in poultry producing states	0.47***	0.70***	-0.02	0.35**
	(0.03)	(0.03)	(0.01)	(0.15)
**Religion (Hindu as baseline)**				
Muslim HH	0.48***	0.70***	0.02**	0.92***
	(0.01)	(0.01)	(0.01)	(0.06)
Christian HH	0.11***	0.22***	0.02*	0.52***
	(0.02)	(0.02)	(0.01)	(0.09)
Sikh HH	-0.08	-0.11**	-0.04	-1.19***
	(0.04)	(0.04)	(0.03)	(0.30)
Buddhist HH	-0.12**	0.08*	-0.01	0.37
	(0.04)	(0.04)	(0.02)	(0.01)
Other religions HH	-0.18***	0.01	0.09***	1.07***
	(0.04)	(0.04)	(0.02)	(0.24)
**Caste (Other Upper Castes as baseline)**				
Scheduled Tribes HH	0.40***	0.27***	0.03***	0.04
	(0.02)	(0.02)	(0.01)	(0.08)
Scheduled Castes HH	0.40***	0.35***	0.04***	0.25***
	(0.01)	(0.01)	(0.01)	(0.07)
Other Backward Classes HH	0.21***	0.19***	0.02***	0.09
	(0.01)	(0.00)	(0.01)	(0.06)
States	YES	YES	YES	YES
Observations	101,658	101,658	101,658	101,658

Coefficients are shown, with standard errors in parentheses. *Significant at 5% level. **Significant at 1% level. ***Significant at 0.1% level. Other social groups included but not reported.

Income and price were statistically significant determinants for both the participation and levels of consumption in both 1993–94 and 2011–12. The positive coefficients of income imply that if a household had a higher income, consumed more PCPM chicken and egg than a household with a lower income. In contrast, the price variables had negative coefficients both for participation and consumption intensity.

The coefficients of some of the household compositional characteristics were also significant predictors of the consumption decisions for both rounds and had opposite signs in participation and levels decisions (e.g., HH size, HH head is female, HH have boys below 18 years and children below 5 years), justifying the use of the two-part model. In both rounds, if family size increased by one, the probability of consuming chicken increased, however, conditional on consuming, households with larger families consumed less chicken and egg PCPM quantities than smaller households. This result holds over both periods. Instead, consumption decisions appeared to have changed with respect to a limited number of compositional characteristics variables over a twenty-year period.

The coefficients of the decision to consume show that households with female heads were less likely to consume chicken and eggs, however, conditional on consumption, if a household was headed by a female member, the chicken PCPM consumption level was higher than the PCPM consumption of a household headed by a male in 1993–94, while it was lower in 2011–12. With, eggs, the PCPM consumption level was higher than the PCPM consumption of a household headed by a male both in 1993–94 and 2011–12. Furthermore, the significant coefficients show that households with children below five years old were more likely to consume chicken and eggs in 1993–94. However, conditional on consumption, if in a household there was a child below five years old, the PCPM consumption was lower than the PCPM consumption levels of a household without children. In 2011–12, conditional on consumption, if a household had a child below five, PCPM consumption of eggs changed to positive. Also, for 2011–2012 the significant coefficients showed that households with boys below eighteen years old were more likely to consume chicken and eggs. However, provided consumption, if in a household there was a boy below eighteen years old, chicken and egg PCPM consumption levels were lower than the PCPM consumption of a household without boys. Finally, the consumption decisions across the years changed with the household head’s education. Although not all the coefficients were significant, in 1993–1994 if a household was headed by a head with a higher education level, chicken and eggs PCPM intensity consumption was higher than a household with a head with a lower education. In 2011–12, the consumption participation and intensity coefficients of these variables had an opposite relation with respect to the previous period.

We found that the significant coefficients of being located at the urban level, having primary employment in agriculture, and being located in major poultry-producing states had an important impact on consumption and intensity decisions across both periods. If a household was located at the urban level, chicken PCPM consumption was lower and egg PCPM higher than in a household located in rural areas. Additionally, if a household was employed in the agricultural sector, the PCPM of eggs was lower compared to a household not employed in the agriculture sector for both rounds. Finally, across years, if a household was located in major poultry-producing states, PCPM consumption levels of chicken and eggs were higher compared to PCPM consumption of a household not located in poultry-producing states.

Religion and the social group were also statistically significant determinants for both the participation and intensity of consumption, with the results holding across both sets. Although the magnitude varied across religious groups, provided consumption, if a household was Muslim or Christian, the PCPM consumption level was significantly higher compared to a household that pertained to the Hindu religion across both periods. Likewise, if a household was part of the Sikh religion, chicken, and egg PCPM consumption was significantly lower compared to a Hindu household. As per the social group effects on consumption, in both rounds, if a household pertained to more marginalised caste groups, chicken PCPM consumption levels were higher compared to the PCPM consumption of a household that pertained to the Other/ Upper Castes groups. However, with eggs, in 1993–94, if a household was part of more marginalised caste groups, PCPM intensity consumption was lower in tribal groups than the PCPM consumption of a household from other caste groups, while in 2011–12, if a household pertained to more marginalised groups, eggs PCPM levels of consumption were higher compared to a household from other/ upper castes groups.

### Unconditional quantile regression estimates across quantiles of chicken and egg consumption distribution by household socio-demographic characteristics

Finally, Tables 11 and 12 present results from UQR. In accordance with previous models, PCPM consumption for both items increased with income. The coefficients attached to the income variable increase along with the consumption distribution for both chicken and eggs. As the distribution moves towards higher quantiles, the price covariate, negative at the lower end of the distribution, increases consistently and becomes positive at the higher end for eggs only. Also, households with boys below eighteen years were associated with a decrease in PCPM consumption for both chicken meat and eggs as the distribution moves towards higher quantiles, making this variable more important for households with little consumption of chicken and eggs.

**Table 11:** Unconditional quantile regression estimates for chicken

Quantiles of the chicken consumption (kg PCPM)					
	10^th^	25^th^	50^th^	75^th^	90^th^
Log monthly per-capita expenditure	0.041***	0.063***	0.105***	0.138***	0.303***
	(0.002)	(0.002)	(0.002)	(0.003)	(0.007)
District price	-0.001***	-0.001***	-0.001***	-0.001***	-0.002***
	(0.000)	(0.000)	(0.000)	(0.000)	(0.000)
HH size	-0.008***	-0.012***	-0.011***	-0.020***	-0.024***
	(0.000)	(0.000)	(0.001)	(0.001)	(0.002)
HH head is female	0.003	-0.006*	-0.002	-0.017***	-0.026**
	(0.002)	(0.002)	(0.003)	(0.004)	(0.009)
HH has children below 5 years	-0.005**	-0.014***	-0.001	-0.002	0.010
	(0.002)	(0.002)	(0.002)	(0.003)	(0.007)
HH has boys below 18 years	0.006**	-0.001	-0.010***	-0.052***	-0.067***
	(0.002)	(0.002)	(0.002)	(0.003)	(0.008)
HH head is illiterate	0.005**	0.002	0.008**	0.015***	0.055***
	(0.002)	(0.002)	(0.002)	(0.003)	(0.008)
HH head has primary	0.002	-0.000	0.008**	0.005	0.015
education	(0.002)	(0.002)	(0.003)	(0.004)	(0.009)
HH head has secondary	0.001	0.007**	0.009**	0.002	0.000
education	(0.002)	(0.002)	(0.003)	(0.004)	(0.009)
HH head has university	-0.006*	-0.004	-0.010*	-0.014**	-0.020
education	(0.003)	(0.003)	(0.004)	(0.005)	(0.012)
Urban HH	-0.008***	-0.012***	-0.007**	-0.010**	-0.020**
	(0.002)	(0.002)	(0.002)	(0.003)	(0.007)
Agricultural HH	0.007***	0.006**	0.009***	0.003	-0.004
	(0.002)	(0.002)	(0.002)	(0.003)	(0.008)
HH in poultry producing states	-0.018*	-0.009	-0.030**	0.010	-0.004
	(0.007)	(0.008)	(0.010)	(0.013)	(0.030)
**Religion (Hindu as baseline)**					
Muslim HH	0.000	0.008**	0.013***	0.021***	0.046***
	(0.002)	(0.002)	(0.003)	(0.004)	(0.009)
Christian HH	-0.010	0.003	0.002	0.015*	0.090***
	(0.004)	(0.004)	(0.005)	(0.007)	(0.016)
Sikh HH	0.007	0.003	0.007	0.009	0.016
	(-0.011)	(0.012)	(0.015)	(0.020)	(0.045)
Buddhist HH	-0.003	0.009	0.041***	0.032*	0.055
	(0.008)	(0.008)	(0.010)	(0.014)	(0.032)
Other religions HH	0.013	0.017	0.044*	0.075**	0.269***
	(0.013)	(0.014)	(0.018)	(0.024)	(0.054)
**Caste (Other Upper Castes as baseline)**					
Scheduled Tribes HH	0.017***	0.008**	0.019***	0.023***	0.069***
	(0.003)	(0.003)	(0.004)	(0.005)	(0.012)
Scheduled Castes HH	-0.000	-0.007**	0.002	0.005	0.026**
	(0.002)	(0.002)	(0.003)	(0.004)	(0.009)
Other Backward Classes HH	0.001	0.001	0.004	0.001	0.030***
	(0.002)	(0.002)	(0.003)	(0.003)	(0.008)
States	YES	YES	YES	YES	YES
Observations	41,001	41,001	41,001	41,001	41,001
R squared	0.078	0.152	0.172	0.196	0.132

Coefficients are shown with standard errors in parentheses. *Significant at 5% level. **Significant at 1% level. ***Significant at 0.1% level. Other social groups included but not reported.

**Table 12:** Unconditional quantile regression estimates for eggs

Quantiles of the egg consumption (n PCPM)					
	10^th^	25^th^	50^th^	75^th^	90^th^
Log monthly per-capita expenditure	0.492***	0.565***	1.153***	2.067***	4.345***
	(0.017)	(0.016)	(0.022)	(0.038)	(0.097)
District price	-0.100**	-0.078*	0.034	0.368***	0.726***
	(0.035)	(0.033)	(0.045)	(0.079)	(0.202)
HH size	-0.113***	-0.150***	-0.194***	-0.282***	-0.426***
	(0.005)	(0.004)	(0.006)	(0.010)	(0.026)
HH head is female	0.086***	0.140***	0.231***	0.303***	0.610***
	(0.024)	(0.022)	(0.031)	(0.053)	(0.136)
HH has children below 5 years	-0.041*	-0.050**	0.006	0.116**	0.043
	(0.018)	(0.017)	(0.023)	(0.040)	(0.102)
HH has boys below 18 years	0.021	-0.040*	-0.263***	-0.699***	-1.070***
	(0.020)	(0.019)	(0.026)	(0.045)	(0.116)
HH head is illiterate	-0.027	-0.080***	-0.060*	0.038	0.211
	(0.020)	(0.019)	(0.026)	(0.045)	(0.116)
HH head has primary	0.051*	0.012	0.040	-0.080	0.218
education	(0.024)	(0.022)	(0.031)	(0.054)	(0.138)
HH head has secondary	0.031	0.021	-0.017	-0.065	0.110
education	(0.023)	(0.021)	(0.030)	(0.051)	(0.131)
HH head has university	-0.061	-0.004	-0.126**	-0.087	0.440*
education	(0.032)	(0.030)	(0.041)	(0.072)	(0.184)
Urban HH	0.064**	0.094***	0.142***	0.283***	0.682*
	(0.019)	(0.018)	(0.025)	(0.043)	(0.110)
Agricultural HH	-0.065**	-0.049*	-0.066*	-0.212***	-0.300*
	(0.021)	(0.020)	(0.027)	(0.047)	(0.120)
HH in poultry producing states	0.050	0.314***	0.537***	0.723***	1.119*
	(0.088)	(0.082)	(0.114)	(0.200)	(0.504)
**Religion (Hindu as baseline)**					
Muslim HH	0.116***	0.193***	0.324***	0.327***	0.669***
	(0.022)	(0.020)	(0.028)	(0.049)	(0.125)
Christian HH	0.140**	0.137***	0.328***	0.280**	0.994***
	(0.042)	(0.039)	(0.054)	(0.094)	(0.239)
Sikh HH	-0.011	-0.051	-0.222	-1.479***	-3.448***
	(0.119)	(0.111)	(0.153)	(0.268)	(0.681)
Buddhist HH	-0.091	-0.219***	-0.046	-0.146	-1.140*
	(0.089)	(0.083)	(0.115)	(0.199)	(0.509)
Other religions HH	0.464**	-0.595***	0.804***	0.057	-0.468
	(0.160)	(0.149)	(0.206)	(0.359)	(0.915)
**Caste (Other Upper Castes as baseline)**					
Scheduled Tribes HH	-0.043	0.074*	0.146**	0.362***	0.747***
	(0.033)	(0.031)	(0.043)	(0.074)	(0.190)
Scheduled Castes HH	0.073***	0.121***	0.134***	0.064	0.434**
	(0.024)	(0.022)	(0.031)	(0.054)	(0.137)
Other Backward Classes HH	0.076***	0.090***	0.065*	0.019	0.300**
	(0.020)	(0.019)	(0.026)	(0.046)	(0.116)
States	YES	YES	YES	YES	YES
Observations	44,295	44,295	44,295	44,295	44,295
R squared	0.094	0.163	0.219	0.209	0.127

Coefficients are shown, with standard errors in parentheses. *Significant at 5% level. **Significant at 1% level. ***Significant at 0.1% level. Other social groups included but not reported.

As in the previous models, for eggs, the female head covariate displays positive coefficients, which increase at the higher end of the distribution with a 0.61 coefficient at the 90^th^ percentile. Likewise, for eggs, the coefficients attached to the covariate household with children below five years old display a negative association at the lower percentiles of the distribution but become positive at the higher end. The covariates of education level also bring additional information. While at the lower end of the distribution, the PCPM consumption is minimal and negative for the head with a university degree, at higher quantiles the coefficients become positive and substantially higher.

We also found that the agricultural household variable coefficients are associated with a lower intake of egg consumption that decreases towards the top half of the distribution, and with a significantly positive intake of chicken meat at lower quantiles that previous models did not show. The urban location coefficient has a positive relationship with PCPM egg consumption, which increases from 0.06 to 0.68 eggs when moving from the 10^th^ to the 90^th^ quantile. Whereas it has a negative relationship with chicken PCPM consumption across the whole distribution, which decreases from -0.8 to -2 kg at the 90^th^ quantile. A negative association between households located in major poultry producing states coefficient and chicken consumption is statistically significant only at the lower quantiles of the distribution. In contrast, with eggs, the relationship is statistically significant across the whole distribution and the coefficients which are small at the lower end of the tail, increase substantially at the higher with a 1.17 coefficient at the 90^th^ percentile.

Households’ religious groups’ coefficients also share similar results with previous estimations. Muslim and Christian religious groups’ coefficients are associated with higher PCPM consumption for both chicken and eggs, and the association increases substantially towards the higher tail of the distribution where higher coefficients are displayed. The coefficients attached to the household social strata are indicative in this model. More marginalised castes coefficients are associated with higher PCPM consumption of chicken, and as the distribution moves towards the end of the tail, the coefficients increase remarkably. Similarly, with eggs, the positive coefficients associated with PCPM egg consumption, suggest that egg consumption was higher amongst more marginalised castes, particularly Scheduled Tribes, than Other Upper Castes. This is particularly true towards the end of the distribution.

## Discussion

### Summary of the results and policy implications

By unpacking demand from a multidisciplinary perspective, where poultry production variables are taken into account alongside household determinants, including gender, class/caste, and religious practices, this paper provides insights on key underlying factors of consumption for chicken and eggs over a twenty-year period in India. Firstly, consumption of chicken and eggs, although increased across two decades, in 2011–12 was still very low and highly unequal across the country. This is especially true for rural households, and households living in North and West zones. Secondly, the results of the regression showed that household income, chicken and egg price, religion, caste, and living in poultry producing states and urban areas for eggs, were important factors for consumption. Finally, the association of chicken and egg consumption with the covariates was found to vary substantially across the consumption distribution. It was shown that the most significant covariates in the models displayed a stronger association towards the top end of the quantile consumption distribution.

The Indian poultry sector has experienced significant and fast changes to its structure and operations in the last two decades. The adoption of the integrated model by a limited number of large commercial producers in southern India was the major change that led to fast growth (Landes et al., 2004; Pica-Ciamarra & Otte, 2010). However, the relevance of these transformations for consumers has been limited and inequal as poultry consumption remains low and large-scale poultry production methods have significant external costs for consumers, including food poisonings and diseases and the development of antimicrobial-resistant microorganisms (Kornel, 2008; NBSO, 2017).

Although poultry is becoming more affordable and more available, many Indians still cannot afford to consume it regularly. The results show how household socio-economic factors such as price and income were important barriers, that hold constant over time, to actual consumption. For both periods analysed, intake was remarkably higher in the South of the country and in poultry producing states where the price of chicken and eggs was lower. Prices were also lower in urban areas. The results show that in twenty years the prices have increased across the country, but were lower in the south with respect to other areas of India, particularly for chicken. This may reflect why chicken has seen a surge in the number of households consuming it. Despite, India's income growth in the last few years, a regional trend with the southern states benefiting more in terms of consumption causing greater regional inequality is suggested. Furthermore, the study shows a clear association between higher consumption of eggs and urbanisation, while with chicken a more limited role of urbanisation was found. Pandey et al. (2020), suggest that urbanisation and income are co-related in India, and urbanisation may indirectly influence food consumption through income (Pandey et al., 2020).

In addition, we found suggestions that demand for poultry has been determined by cultural biases that drove consumers away from meat-based consumption despite income gains. Although poultry consumption is increasing amongst the middle-class and is becoming more accepted, cultural and regional influences, and stigmas from caste, religious faiths and social identity still shape food eating habits in India, particularly for meat (Atkin et al., 2021; Chakravarti, 1974; Ferry, 2020; Khamis et al., 2012). Despite that only 30% of Indians are strictly vegetarians, the average consumption of poultry products remains very low in the country due to cultural, economic, and political reasons (Devi et al., 2014; Sample Registration System, 2014). Non-vegetarian foods continue to be a contentious topic in some schools, workplaces, and religious places, and many Indians, particularly women, experience conflict towards the practice of non-vegetarian food eating (Ahmad, 2018; Bruckert, 2015; Drèze, 2019; Khara et al., 2020). Results support these ideas put forward by the literature. Variables representing cultural and religious beliefs, together with income, emerged as some of the most important factors that may serve as barriers to consumption.

Furthermore, intrahousehold food allocation resulting in different consumption outcomes for women and young children with respect to male members also emerged as having a role in reducing consumption. Households with boys below 18 years consumed more chicken and eggs compared to households without young boys. Households headed by females consumed less chicken meat than households headed by a male. Bruckert (2015) explains that female members and especially the mothers, through their cooking, are the ones in charge of preserving the purity of the household from impure foods (meat) and the ones that are more subject to preserving vegetarian codes within the household (Bruckert, 2015). Iannotti et al. (2014) found that cultural beliefs about egg digestibility or cleanliness, together with concerns about allergies and cholesterol, may inhibit consumption among mothers and children in India and Nepal (Iannotti et al., 2014).

To instil change for improved poultry consumption and production outcomes, government policy has a range of relevant tools that can affect poultry value chain actors, including producers, consumers, and health professionals. This paper’s findings suggest that household income growth and lower prices for eggs and chicken in India increase consumption, particularly amongst more marginalised groups, where cost constraints are the main factors that may limit poultry intake. While a study on fruit and vegetable consumption highlighted Upper castes having a consumption advantage over marginalised groups (Choudhury et al., 2020), with chicken and eggs, we found that Scheduled Tribes, Scheduled Castes, and Other Backward Classes consumed more quantities than Upper Castes. This is an important finding as it highlights the potential to increase protein intake with the part of the population that is the most protein insecure. These groups have been historically marginalised due to poorly targeted policies and also suffer from high levels of hunger and malnutrition (Pillay & Kumar, 2018; Yu et al., 2015).

Given the large protein consumption deficit particularly in rural India and in certain regions of the country, there is a need for more tailored consumer policies for marginalised consumers. For example, nutrition schemes and programs that promote access to affordable and high-quality poultry and eggs have the potential to improve nutritional outcomes, particularly for women and children. Although certain Indian states have restricted access to eggs in the Mid-Day Meal scheme, the scheme with the incorporation of eggs proved to be successful in Tamil Nadu (Drèze, 2019; Maneesh, 2015).

Educational programmes may also encourage change in eating behaviour (Iannotti et al., 2014; Raghunathan et al., 2020). Some nutritional programmes have targeted mothers of young children to improve infant and young children feeding practices with positive impacts (Iannotti et al., 2009; Malhotra, 2013). Efforts to improve consumer knowledge and awareness regarding poultry products’ nutritional values through media campaigns and school-based interventions are needed in collaboration with health professionals and the private sector. It is highlighted that marginalised caste groups sometimes give up on eating non-vegetarian food out of social pressure, either to increase their social status or because they feel guilty for violating vegetarian food norms, particularly women (Khara et al., 2021). Therefore, while simultaneously promoting health, these policies will need to tailor strategies to further de-stigmatise chicken and egg consumption reaching vulnerable groups for improved nutrition.

### Limitations and future research

Several weaknesses associated with the study are recognised. Firstly, there are limitations associated with the use of household surveys to measure food consumption estimates. These include biases and measurement errors owing to systematic and non-sampling errors, the irregularity in the collection of the NSS data (the latest data are not available), and the household level nature of the data that do not reflect the access to food by individuals (Smith, 2002). Specifically, type 1 data of NSS are found to show lower intake for foods and thus possibly lead to underestimated results (Aleksandrowicz et al., 2017). Secondly, weaknesses in the study are due to the use of a demand single equation approach, the choice to use proxies for certain variables (e.g. price, income, and education), and the absence of variables that represent markets, road networks and infrastructures in the regression that may be of importance. Thirdly, the estimates might be affected by an endogeneity problem as consumption of both chicken meat and eggs could be linked to the informal production of poultry in areas that have an absent market. However, given the nationally representative nature of NSSO data, the study provides key insights into heterogeneous poultry consumption practices across two decades in India.

Future research should analyse more recent data on poultry consumption in India. NSS data are usually revised after five years, but the 2015–2016 round never became available. If the latest round becomes available, monitoring the changes in outcomes should be studied. This paper’s datasets do not contain data on market information and infrastructures. In India, cold chain infrastructures are not well developed and poultry products, highly perishable, are not well distributed (Landes et al., 2004; Pica-Ciamarra & Otte, 2010). The analysis of the market can reveal further information on poultry consumption, e.g., whether the household’s proximity to the market can increase per-capita poultry consumption. Questions also arise about how important negative externalities from intensive production such as endemic diseases of poultry in India are perceived by Indian consumers and how policies and interventions to tackle zoonosis and food safety threats from poultry play a role in demand and ecological factors. Fast developments in the poultry sector, market concentration and the associated risks of pandemics and health crises, are likely to influence production and consumption. Having such data would be beneficial to better understand linkages of consumption and production.

## Conclusions

Despite limited knowledge of why there is low consumption of poultry products, especially eggs, in India, evidence for its potential to improve nutrition outcomes is plentiful. This paper finds, in accordance with several studies, that consumption was very low especially at the rural level and in North and West zones of India. We show that PCPM intake was higher in the South where major poultry producers are concentrated and where the products have also been more affordable (Landes et al., 2004; Pica-Ciamarra & Otte, 2010). This suggests that supply-side determinants influence heterogeneous consumption patterns in India.

Our analysis also shows that along with income, household dynamics and socio-cultural characteristics are important factors for consumption. Pertaining to Hindu and Sikh religions and being part of Upper Caste groups reduced eggs and chicken intake. This is in line with existing literature that finds that cultural and religious taboos limit ASFs consumption in India (Ahmad, 2018; Filippini & Srinivasan, 2019; Morris et al., 2018; Simoons, 1994). However, a key result of this paper is that marginalised groups including Other Backward Classes, Scheduled Tribes, and Scheduled Castes consumed more chicken and eggs than Other Upper Castes. Income constraints and higher prices are likely to be the major limit to poultry consumption for these groups.

Another important finding was the gender dimension of consumption. In line with other studies flagging that boys eat more than females in the household (Aurino, 2017), we found that households with boys below 18 years consumed more chicken and eggs compared to households without young boys. Additionally, we found that households headed by females consumed less chicken meat than households headed by a male. Overall, the study highlighted important socio-cultural factors that have been driving poultry consumption. Rapidly changing poultry production systems can generate risks for public health and increase consumption inequalities across the country. These understandings of demand determinants generate reflections to better support the Indian poultry sector policy planning. The findings have policy implications for malnutrition in the country, as the promise of high annual growth rates and lower food conversion ratios from the poultry industry may not translate to improved nutrition for all.

## Data Availability

The data analysed in this study is subject to the following licenses: Data sets are not available in the public domain but can be purchased on request from the National Sample Survey Office (NSSO) and the Central Statistics Office (CSO), Government of India. Requests to access these datasets should be directed to http://www.mospi.gov.in/nsso; https://www.cso.ie/en/index.html.

## Footnotes

1 The 50th round of NSSO does not report districts, but has a concept of State-Region described as a contiguous group of districts within a State having similar topography, agro-economic characteristics, and population densities.

2 Round 50^th^ and previous rounds of NSSO data do not have the “Other Backward Classes” category.

3 Urban area is defined as “a) all places with a Municipality, Corporation or Cantonment and places notified as town area b) all other places which satisfied the following criteria: (i) a minimum population of 5000, (ii) at least 75% of the male working population are non-agriculturists, and (iii) a density of population of at least 1000 per sq. mile (390 per sq. km.). There are urban areas which do not possess all the above characteristics uniformly. Certain areas were treated as urban on the basis of their possessing distinct urban characteristics, overall importance and contribution to the urban economy of the region”.

## References

[CR1] Adesogan, A. T., Havelaar, A. H., McKune, S. L., Eilittä, M., & Dahl, G. E. (2020). Animal source foods: Sustainability problem or malnutrition and sustainability solution? Perspective matters. In *Global Food Security,* *25*. 10.1016/j.gfs.2019.100325

[CR2] Ahmad Z (2018). Delhi’s Meatscapes Muslim Butchers in a Transforming Mega City.

[CR3] Aleksandrowicz, L., Tak, M., Green, R., Kinra, S., & Haines, A. (2017). Comparison of food consumption in Indian adults between national and sub-national dietary data sources. *British Journal of Nutrition*, *117*(7). 10.1017/S0007114517000563 10.1017/S0007114517000563 28462737

[CR4] Amugsi, D. A., Lartey, A., Kimani, E., & Mberu, B. U. (2016). Women’s participation in household decision-making and higher dietary diversity: findings from nationally representative data from Ghana. *Journal of Health, Population, and Nutrition*, *35*(1). 10.1186/s41043-016-0053-1 10.1186/s41043-016-0053-1 PMC5026004 27245827

[CR5] Atkin, D., Colson-Sihra, E., & Shayo, M. (2021). How do we choose our identity? a revealed preference approach using food consumption. *Journal of Political Economy*, *129*(4), 1193–1251.

[CR6] Aurino, E. (2017). Do boys eat better than girls in India? Longitudinal evidence on dietary diversity and food consumption disparities among children and adolescents. *Economics and Human Biology*, *25*. 10.1016/j.ehb.2016.10.007 10.1016/j.ehb.2016.10.007 27810442

[CR7] Balli, H. O., Kouhbor, M. A., & Jean Louis, R. (2017). Towards Understanding Vegetables Consumption Behaviour in Iran: A Full Box-Cox Double-Hurdle Application. *Review of Middle East Economics and Finance*, *13*(1). 10.1515/rmeef-2016-0017

[CR8] Bruckert M (2015). Une géographie de la viande au Tamil Nadu (Inde): Statuts, espaces et circulations.

[CR9] Bruckert, M. (2021). Chicken Politics: Agrifood Capitalism, Anxious Bodies, and the New Meanings of Chicken Meat in India. *Gastronomica*.

[CR10] Burton, M., Tomlinson, M., & Young, T. (1994). Consumers‘ decisions whether or not to purchase meat: a double hurdle analysis of single adult households. *Journal of Agricultural Economics*, *45*(2). 10.1111/j.1477-9552.1994.tb00394.x

[CR11] Chakravarti AK (1974). Regional Preference for Food: Some Aspects of Food Habit Patterns in India. Canadian Geographer/le Géographe Canadien.

[CR12] Choudhury, S., Shankar, B., Aleksandrowicz, L., Tak, M., Green, R., Harris, F., Scheelbeek, P., & Dangour, A. (2020). What underlies inadequate and unequal fruit and vegetable consumption in India? An exploratory analysis. *Global Food Security*, *24*. 10.1016/j.gfs.2019.100332 10.1016/j.gfs.2019.100332 PMC7063694 32190538

[CR13] DAHD. (2012). *Indian Production of Poultry*. Retrieved on February 13, 2023 from https://agriexchange.apeda.gov.in/India%20Production/AgriIndia_Productions.aspx?productcode=1024

[CR14] DAHD. (2020). *Basic animal husbandry statistics 2020*.

[CR15] Das, D. K. (2016). Book Review: Rajesh Raj S. N. and Kunal Sen. 2016. Out of the Shadows? The Informal Sector in Post-reform India. *Journal of South Asian Development*, *11*(3). 10.1177/0973174116676694

[CR16] Deaton A (1988). Quality, Quantity, and Spatial Variation of Price. American Economics Review.

[CR17] Deloitte. (2018). *Industry 4.0 in Food Industry-India Food Report. India*.

[CR18] Devi SM, Balachandar V, Lee SI, Kim IH (2014). An outline of meat consumption in the indian population-A pilot review. In Korean Journal for Food Science of Animal Resources.

[CR19] Dolphijn, R. (2006). Capitalism on a Plate: The Politics of Meat Eating in Bangalore, India. *Gastronomica*, *6*(3). 10.1525/gfc.2006.6.3.52

[CR20] Drèze, J. (2019). School Meals. In *Sense and solidarity: Jholawala economics for everyone.* (pp. 69–71). Oxford University Press.

[CR21] Ferry M (2020). What’s India’s beef with meat? Hindu orthopraxis and food transition in India since the 1980s. Sociological Forum.

[CR22] Filippini, M., & Srinivasan, S. (2019). Impact of religious participation, social interactions and globalization on meat consumption: Evidence from India. *Energy Economics*, *84*. 10.1016/j.eneco.2019.104550

[CR23] Firpo, S., Fortin, N. M., & Lemieux, T. (2009). Unconditional quantile regressions S Firpo, NM Fortin, T Lemieux - Econometrica, 2009. *Econometrica*.

[CR24] García, J., & Labeaga, J. M. (1996). Alternative approaches to modelling zero expenditure: An application to Spanish demand for tobacco. *Oxford Bulletin of Economics and Statistics*, *58*(3). 10.1111/j.1468-0084.1996.mp58003004.x

[CR25] GOI. (2014). *National Sample Survey Office (2014) Household Consumption of Various Goods and Services in India 2011–12. NSS 68th Round.*

[CR26] Hoddinott, J., & Haddad, L. (1995). Does female income share infleunce household expenditures? Evidence from Côte d’Ivoire. *Oxford Bulletin of Economics and Statistics*, *57*(1). 10.1111/j.1468-0084.1995.tb00028.x

[CR27] Iannotti, L., Cunningham, K., Ruel, M., Spielman, D., & Pandya-Lorch, R. (2009). Diversifying into healthy diets: homestead food production in Bangladesh. *Millions Fed: Proven Successes in Agricultural Development*.

[CR28] Iannotti, L., Lutter, C. K., Bunn, D. A., & Stewart, C. P. (2014). Eggs: The uncracked potential for improving maternal and young child nutrition among the world’s poor. *Nutrition Reviews*, *72*(6). 10.1111/nure.12107 10.1111/nure.12107 24807641

[CR29] ICFA. (n.d.). *Indian Poultry Market Overview*. Retrieved on January 30, 2021 from https://www.icfa.org.in/assets/doc/reports/Indian_Poultry_Market_Overview.pdf

[CR30] Kennedy, E., & Peters, P. (1992). Household food security and child nutrition: the interaction of income and gender of household head. *World Development*, *20*(8). 10.1016/0305-750X(92)90001-C

[CR31] Khamis M, Nishith P, Zahra S (2012). Consumption and social identity: Evidence from India. Journal of Economic Behavior & Organization.

[CR32] Khan, M. A., & Ravichandran, P. (2015). A Study on Influencing Factors to Affect the Economic Status of Layer Poultry Farmers in Namakkal District of Tamilnadu. *International Journal of Economics and Management Sciences*, *04*(04). 10.4172/2162-6359.1000242

[CR33] Khara, T., Riedy, C., & Ruby, M. B. (2020). “We have to keep it a secret” – The dynamics of front and backstage behaviours surrounding meat consumption in India. *Appetite*, *149*. 10.1016/j.appet.2020.104615 10.1016/j.appet.2020.104615 31996317

[CR34] Khara, T., Riedy, C., & Ruby, M. B. (2021). A cross cultural meat paradox: A qualitative study of Australia and India. *Appetite*, *164*. 10.1016/j.appet.2021.105227 10.1016/j.appet.2021.105227 33812938

[CR35] Kornel D (2008). Poultry sector country review.

[CR36] Kumar, N., & Kapoor, S. (2014). Study of consumers’ behavior for non-vegetarian products in emerging market of India. *Journal of Agribusiness in Developing and Emerging Economies*, *4*.

[CR37] Kumar, P., Joshi, P. K., & Mittal, S. (2016). Demand vs supply of food in India - Futuristic projection. *Proceedings of the Indian National Science Academy*. 10.16943/ptinsa/2016/48889

[CR38] Lagos, J. E., & Intodia, V. (2015). *Poultry and Poultry Products Annual 2015. GAIN report.* https://apps.fas.usda.gov/newgainapi/api/report/downloadreportbyfilename?filename=Poultry and Poultry Products Annual 2015_New Delhi_India_9–30–2015.pdf

[CR39] Lancaster, G., Ray, R., & Maitra, P. (2006). Gender Bias in Nutrient Intake: Evidence from Selected Indian States. *South Asia Economic Journal*, *7*(2). 10.1177/139156140600700206

[CR40] Landes, M., Persaud, S. C., & Dyck, J. (2004). *India’s Poultry Sector: Development and Prospects. Agriculture and Trade Report No. WRS04–03.* Retrieved on January 30, 2021 from https://www.ers.usda.gov/publications/pub-details/?pubid=40407

[CR41] Lung, W. F., Yong, K. C., & Abdul Razak, N. A. (2020). Sociodemographic Factors as Determinants of Fruit and Vegetable Consumption in Malaysia. *Jurnal Sains Kesihatan Malaysia*, *18*(02). 10.17576/jskm-2020-1802-03

[CR42] MAFW. (2017). *National Action Plan for Egg & Poultry-2022 For Doubling Farmers’ Income by 2022*. Retrieved on February 13, 2023 from https://www.dahd.nic.in/sites/default/filess/Seeking%20Comments%20on%20National%20Action%20Plan-%20Poultry-%202022%20by%2012-12-2017.pdf

[CR43] Maitra P, Rammohan A, Ray R, Robitaille MC (2013). Food consumption patterns and malnourished Indian children: Is there a link?. Food Policy.

[CR44] Malhotra N (2013). Inadequate feeding of infant and young children in India: Lack of nutritional information or food affordability?. Public Health Nutrition.

[CR45] Maneesh, P. (2015). Mid Day Meals and Food Security among Children: Assuring Nutritional Security of Tamil Nadu. *Indian Journal of Economics and Development*, *3*(9).

[CR46] Mehraban, N., & Ickowitz, A. (2021). Dietary diversity of rural Indonesian households declines over time with agricultural production diversity even as incomes rise. *Global Food Security*, *28*. 10.1016/j.gfs.2021.100502

[CR47] Minocha, S., Thomas, T., & Kurpad, A. v. (2018). Are “fruits and vegetables” intake really what they seem in India? *European Journal of Clinical Nutrition*, *72*(4). 10.1038/s41430-018-0094-1 10.1038/s41430-018-0094-1 29459786

[CR48] Mishra, A. K., Mottaleb, K. A., & Mohanty, S. (2015). Impact of off-farm income on food expenditures in rural Bangladesh: An unconditional quantile regression approach. *Agricultural Economics (United Kingdom)*, *46*(2). 10.1111/agec.12146

[CR49] Morris, S. S., Beesabathuni, K., & Headey, D. (2018). An egg for everyone: Pathways to universal access to one of nature’s most nutritious foods. *Maternal and Child Nutrition*, *14*. 10.1111/mcn.12679 10.1111/mcn.12679 PMC6866008 30332534

[CR50] Nanda Kumar T, Samantara A, Gulati A (2022). Poultry Value Chain..

[CR51] National Institute of Nutrition. (2011). *Dietary Guidelines for Indians* .

[CR52] NBSO. (2017). *Poultry Sector Opportunities and Challenges in India*.

[CR53] NSSO. (2014). *Nutritional intake in India, 2011–12 NSS 68th Round, Report No. 560.* .

[CR54] Pandey, B., Reba, M., Joshi, P. K., & Seto, K. C. (2020). Urbanization and food consumption in India. *Scientific Reports*, *10*(1). 10.1038/s41598-020-73313-8 10.1038/s41598-020-73313-8 PMC7560883 33057014

[CR55] Pica-Ciamarra U, Otte J (2010). Poultry, food security and poverty in India: Looking beyond the farm-gate. In World’s Poultry Science Journal.

[CR56] Pillay DPK, Kumar TKM (2018). Food security in India: Evolution, efforts and problems. Strategic Analysis.

[CR57] Pingali, P., & Khwaja, Y. (2004). Globalisation of Indian Diets and the Transformation of Food Supply Systems. *Indian Journal of Agricultural Marketing*, *18*(1).

[CR58] Raghunathan K, Headey D, Herforth A (2020). Affordability of nutritious diets in rural India. Food Policy.

[CR59] Raskind, I. G., Patil, S. S., Haardörfer, R., & Cunningham, S. A. (2018). Unhealthy Weight in Indian Families: The Role of the Family Environment in the Context of the Nutrition Transition. *Population Research and Policy Review*, *37*(2). 10.1007/s11113-017-9455-z 10.1007/s11113-017-9455-z PMC6023548 29962562

[CR60] Rautela, G., Ali, M. K., Prabhakaran, D., Narayan, K. M. V., Tandon, N., Mohan, V., & Jaacks, L. M. (2020). Prevalence and correlates of household food insecurity in Delhi and Chennai, India. *Food Security*, *12*(2). 10.1007/s12571-020-01015-0 10.1007/s12571-020-01015-0 PMC7810060 33456633

[CR61] Rios-Avila, F. (2020). Recentered influence functions (RIFs) in Stata: RIF regression and RIF decomposition. *Stata Journal*, *20*(1). 10.1177/1536867X20909690

[CR62] Saha, S., Vemula, S. R., Mendu, V. V. R., & Gavaravarapu, S. R. M. (2013). Knowledge and practices of using food label information among adolescents attending schools in Kolkata, India. *Journal of Nutrition Education and Behavior*. 10.1016/j.jneb.2013.07.011 10.1016/j.jneb.2013.07.011 24021455

[CR63] Sample Registration System. (2014). *Sample Registration System Baseline Survey 2014*.

[CR64] Sathyamala, C. (2019). Meat-eating in India: Whose food, whose politics, and whose rights? *Policy Futures in Education*, *17*(7). 10.1177/1478210318780553

[CR65] Sharma, M., Kishore, A., Roy, D., & Joshi, K. (2020). A comparison of the Indian diet with the EAT-Lancet reference diet. *BMC Public Health*, *20*(1). 10.1186/s12889-020-08951-8 10.1186/s12889-020-08951-8 PMC7260780 32471408

[CR66] Simoons, F. J. (1994). Eat not this flesh. In *Food avoidances from prehistory to the present (2nd ed.).* (Issue 3). Madison, Wisconsin, U.S.A: University of Wisconsin Press.

[CR67] Smith, L. C. (2002). Keynote Paper: The use of household expenditure surveys for the assessment of food insecurity. In *Measurement and Assessment of Food Deprivation and Undernutrition*.

[CR68] Smith L, Subandoro A (2007). Measuring Food Security Using Household Expenditure Surveys. In Measuring Food Security Using Household Expenditure Surveys.

[CR69] StataCorp.  (2015). Stata Statistical Software: Release 14.

[CR70] Tak, M., Shankar, B., & Kadiyala, S. (2019). Dietary Transition in India: Temporal and Regional Trends, 1993 to 2012. *Food and Nutrition Bulletin*, *40*(2). 10.1177/0379572119833856 10.1177/0379572119833856 31006264

[CR71] Tefft J, Jonasova M, Adjao R, Morgan A (2017). Food Systems for an Urbanizing World. In Food Systems for an Urbanizing World.

[CR72] Yu W, Elleby C, Zobbe H (2015). Food security policies in India and China: Implications for national and global food security. Food Security.

[CR73] Zaharia, S., Ghosh, S., Shrestha, R., Manohar, S., Thorne-Lyman, A. L., Bashaasha, B., Kabunga, N., Gurung, S., Namirembe, G., Appel, K. H., Liang, L., & Webb, P. (2021). Sustained intake of animal-sourced foods is associated with less stunting in young children. *Nature Food*, *2*(4). 10.1038/s43016-021-00259-z 10.1038/s43016-021-00259-z 37118465

[CR74] Zanello, G., Srinivasan, C. S., & Shankar, B. (2016). What explains Cambodia’s success in reducing child stunting-2000–2014? *PLoS ONE*, *11*(9). 10.1371/journal.pone.0162668 10.1371/journal.pone.0162668 PMC5029902 27649080

[CR75] Zhang, Z., Goldsmith, P. D., & Winter-Nelson, A. (2016). The Importance of Animal Source Foods for Nutrient Sufficiency in the Developing World: The Zambia Scenario. *Food and Nutrition Bulletin*, *37*(3). 10.1177/0379572116647823 10.1177/0379572116647823 27150300

